# Sleep Disturbance at Cancer Diagnosis and Its Associations With Depressive Symptoms and Systemic Inflammatory Burden

**DOI:** 10.1002/pon.70576

**Published:** 2026-08-11

**Authors:** Zeynep Gülsüm Güç, Hüseyin Döngelli, Hasan Güç, Ahmet Alacacıoğlu, Mustafa Oktay Tarhan

**Affiliations:** ^1^ Department of Medical Oncology Atatürk Training and Research Hospital İzmir Katip Çelebi University İzmir Türkiye; ^2^ Department of Internal Medicine Buca Seyfi Demirsoy Training and Research Hospital İzmir Türkiye; ^3^ Department of Plastic Reconstructive and Aesthetic Surgery İzmir Tepecik Training and Research Hospital İzmir Türkiye; ^4^ Department of Medical Oncology Institute of Oncology Dokuz Eylül University İzmir Türkiye

**Keywords:** cancer diagnosis, depression, inflammatory biomarkers, psycho‐oncology, sleep disturbance, systemic inflammation

## Abstract

**Background:**

Sleep disturbance is highly prevalent among cancer patients and closely associated with depressive symptoms. However, it remains unclear whether sleep problems present at cancer diagnosis primarily reflect depression or also indicate an underlying biological susceptibility.

**Aim:**

This study aimed to examine the relationships between sleep disturbance, depressive symptoms, and systemic inflammatory–nutritional burden in newly diagnosed, treatment‐naïve cancer patients.

**Methods:**

In this cross‐sectional study, sleep quality was assessed using the Pittsburgh Sleep Quality Index (PSQI) in patients at diagnosis before oncological treatment. Poor sleep quality (PSQ) was defined as PSQI ≥ 5, and sleep disturbance severity was evaluated using the total PSQI score. Depressive symptoms were measured with the Hospital Anxiety and Depression Scale–Depression subscale (HADS‐D). Systemic inflammatory indices, including neutrophil‐to‐lymphocyte ratio (NLR) and lactate dehydrogenase–to‐albumin ratio (LAR), were calculated from routine laboratory data. Multivariable logistic regression and generalized linear models assessed factors associated with PSQ and sleep disturbance severity, respectively. Receiver operating characteristics (ROC) analyses evaluated the discriminatory performance of depressive symptoms and inflammatory indices. Stratified analyses were conducted according to depression status (HADS‐D < 8 vs. ≥ 8).

**Results:**

Sleep disturbance was highly prevalent at diagnosis. Depressive symptom burden was independently associated with both PSQ and higher PSQI scores. Importantly, systemic inflammatory indices remained independently associated with sleep disturbance after adjustment for depressive symptoms and clinical variables. NLR showed moderate discriminatory ability for PSQ, while LAR was independently associated with sleep disturbance severity. ROC analyses indicated that depressive symptoms and inflammatory burden jointly improved discrimination of PSQ. In stratified analyses, LAR remained significantly associated with PSQI scores regardless of depression status.

**Conclusion:**

Sleep disturbance at cancer diagnosis is independently associated with both depressive symptoms and systemic inflammatory–nutritional burden, suggesting that it may reflect not only psychological distress but also underlying biological processes. Routine sleep assessment alongside inflammatory markers may help identify vulnerable patients and support early supportive care planning.

## 1 Introduction

Sleep disturbance is among the most commonly reported symptoms in cancer populations and represents a major contributor to impaired quality of life (QoL). It often emerges at the time of diagnosis and persists throughout the course of the disease [1, 2]. Poor sleep quality (PSQ), insomnia symptoms, and nocturnal awakenings have been frequently reported even at the time of cancer diagnosis, prior to initiation of oncological treatment [3, 4]. Beyond its impact on QoL, sleep disturbance is associated with significant morbidity and functional impairment [5]. Depressive symptoms are also highly prevalent around the time of diagnosis and represent one of the most common psychological challenges during this period [6].

Sleep disturbance and depression frequently co‐occur and share a bidirectional relationship. Sleep problems increase the risk of developing depressive symptoms, while depression may further exacerbate sleep disturbance [1, 7]. Contemporary psycho‐oncology frameworks increasingly conceptualize sleep disturbance not only as a consequence of depression but also as an independent behavioral process that may contribute to the onset and maintenance of depressive symptoms [8]. From a clinical perspective, sleep disturbances may occur as primary disorders or as secondary manifestations of psychological or medical conditions. According to contemporary diagnostic frameworks, including DSM‐5 classifications, insomnia can present as a distinct disorder but more commonly arises in association with factors such as depression, anxiety, chronic illness, or systemic physiological stress. In cancer populations, they are often interpreted as responses to emotional distress or disease burden; however, emerging biobehavioral models suggest that sleep disturbance may also reflect interactions between psychological and biological pathways.

Growing evidence indicates that sleep disturbance is associated with systemic inflammation. Studies in both general and clinical populations have demonstrated links between PSQ and elevated proinflammatory markers such as C‐reactive protein (CRP), interleukin‐6 (IL‐6), and tumor necrosis factor‐α (TNF‐α) [9, 10]. Meta‐analyses further suggest that sleep disturbance is related to inflammatory activation independent of sleep duration [11]. In cancer, inflammation is a central biological process and has been implicated in behavioral comorbidities including fatigue, depression, and sleep disturbance [1, 12].

Psychoneuroimmunological models propose that sleep disturbance may activate inflammatory pathways, while inflammation may in turn reinforce depressive symptoms and sleep disruption [1, 13]. However, it remains unclear whether the association between sleep disturbance and systemic inflammation in cancer patients is independent of depressive symptoms.

Most previous psycho‐oncology studies examining these relationships have involved relatively small samples, focused primarily on cytokine levels, and were conducted during or after cancer treatment [14, 15, 16]. The diagnostic phase represents a distinct clinical period in which tumor‐related biological processes are active while treatment‐related influences—such as corticosteroid exposure, chemotherapy toxicities, and symptom escalation—have not yet emerged. Investigating sleep disturbance during this early phase may therefore allow assessment in a relatively less confounded biological and psychological context. Biobehavioral data from this early phase is limited but of considerable clinical and scientific importance [3, 17].

Recently, composite inflammatory indices derived from routine laboratory tests—such as the neutrophil‐to‐lymphocyte ratio (NLR), systemic immune‐inflammation index (SII), systemic inflammation response index (SIRI), and lactate dehydrogenase‐to‐albumin ratio (LAR)—have been proposed as accessible markers of systemic inflammatory status [18, 19]. Although these indices have been associated with psychological symptoms such as depression and anxiety [20, 21], their relationship with sleep disturbance in treatment‐naïve cancer patients has not been well characterized.

Therefore, this study aimed to examine the association between sleep disturbance and systemic inflammatory indices in newly diagnosed cancer patients prior to treatment. Specifically, we sought to evaluate whether inflammatory markers are significantly associated with sleep quality and whether these associations remain independent of depressive symptom burden and identify inflammatory markers most strongly associated with sleep disturbance.

## 2 Materials and Methods

### 2.1 Study Design and Patient Population

This single‐center cross‐sectional observational study included 738 patients with histopathologically confirmed solid tumors who were referred to the Medical Oncology Clinic of İzmir Katip Çelebi University Atatürk Training and Research Hospital in 2024–2025. All assessments were conducted after cancer diagnosis and prior to the initiation of any oncological treatment, including chemotherapy, radiotherapy, hormonotherapy, or immunotherapy, to minimize treatment‐related biological and psychological confounding. Patients with a planned systemic treatment strategy were considered treatment‐naïve at the time of evaluation.

### 2.2 Inclusion and Exclusion Criteria

Eligible participants were adults (≥ 18 years) with a histopathologically confirmed solid tumor who had not yet received any systemic or local anticancer treatment, were able to reliably complete psychometric questionnaires and accepted to be included in the study. Patients with acute infection, active inflammatory or autoimmune disease, chronic corticosteroid or immunosuppressive use, or a history of major psychiatric disorders (e.g., psychotic disorders, bipolar disorder, or active substance use disorder) were excluded.

### 2.3 Clinical and Demographic Data

Demographic and clinical characteristics, including age, sex, cancer type, and disease stage, were retrieved from electronic medical records. Tumor stage was determined according to the Tumor–Node–Metastasis (TNM) classification of the American Joint Committee on Cancer and categorized as localized, locally advanced, or metastatic.

### 2.4 Assessment of Sleep Quality

Sleep quality was evaluated using the Pittsburgh Sleep Quality Index (PSQI), a validated self‐report instrument assessing sleep over the previous month (total score 0–21). PSQ was defined as PSQI ≥ 5. For analysis, PSQI was treated both as a continuous variable (total score) and as a categorical variable (good vs. poor sleep quality).

### 2.5 Assessment of Depressive Symptoms

Depressive symptoms were assessed using the depression subscale of the Hospital Anxiety and Depression Scale (HADS‐D), a validated instrument designed for medically ill populations. Scores range from 0 to 21, with clinically significant depressive symptoms defined as HADS‐D ≥ 8. HADS‐D was analyzed as both a continuous and categorical variable. This approach allowed the assessment of both depressive symptom severity and clinically meaningful depression within the statistical models.

### 2.6 Laboratory Measurements and Inflammatory Indices

Laboratory data were obtained from routine blood samples collected concurrently with psychometric questionnaire administration and prior to treatment initiation. Complete blood counts, including neutrophil, lymphocyte, monocyte, and platelet counts, and biochemical parameters, including albumin and lactate dehydrogenase (LDH), were measured using standard methods.

The following systemic inflammatory indices were calculated: NLR, SII (neutrophil × platelet/lymphocyte), SIRI (neutrophil × monocyte/lymphocyte), and LAR (LDH/albumin). Variables with right‐skewed distributions were log‐transformed prior to analysis.

Prior to analysis, the distributions of inflammatory indices were examined. Variables exhibiting right‐skewed distributions were log‐transformed, as appropriate, to meet the assumptions of parametric statistical analyses.

### 2.7 Statistical Analysis

Statistical analyses were performed using IBM SPSS Statistics (IBM Corp., Armonk, NY, USA). Distributional assumptions for continuous variables were assessed using the Kolmogorov–Smirnov test and visual inspection of histograms. As most continuous variables exhibited non‐normal, right‐skewed distributions, data were summarized as mean ± standard deviation or median (IQR), as appropriate.

Baseline sociodemographic, clinical, and laboratory characteristics were compared between patients with good sleep quality (GSQ) and PSQ, defined as PSQI < 5 and ≥ 5, respectively. Group comparisons were performed using independent samples *t* test or Mann–Whitney *U* test for continuous variables and chi‐square or Fisher's exact test for categorical variables. PSQI component scores and total scores were summarized and compared between groups to characterize sleep‐related differences.

Factors associated with PSQ (PSQI ≥ 5) were evaluated using binary logistic regression. Univariable analyses were initially conducted for candidate variables. Variables with *p* < 0.10 and clinically relevant covariates were entered into the multivariable model using the enter method. Multicollinearity among independent variables was assessed using variance inflation factor (VIF) and highly correlated variables were not included simultaneously in the same model.

Model performance was evaluated using the Omnibus test of model coefficients, Nagelkerke *R* ^*2*^, classification accuracy. Model calibration was initially assessed using the Hosmer–Lemeshow goodness‐of‐fit test, acknowledging its sensitivity in large samples. Results were reported as odds ratios (ORs) with 95% confidence intervals (CIs). Internal validation of the multivariable model was performed using bootstrap resampling (1000 iterations) to estimate potential optimism in model performance. Discriminative performance of selected clinical, psychological, and inflammatory–nutritional markers, as well as the multivariable logistic regression model, for identifying PSQ was assessed using a non‐parametric approach. Optimal cut‐off values were determined based on the Youden index, with associated sensitivity and specificity values reported. Cut‐off values derived from ROC analyses were intended to reflect statistical discrimination rather than clinical diagnostic thresholds.

Sensitivity analyses were performed to assess whether the near‐perfect discriminative performance of LAR was influenced by extreme values. Outliers were identified based on standardized residuals (> 3) from the logistic regression model and extreme predicted probabilities (0 or 1). ROC analyses were then repeated using log‐transformed LAR and after excluding identified outliers to evaluate the robustness of the predictive performance.

To further examine the determinants of sleep quality as a continuous outcome, generalized linear models (GLMs) were fitted with PSQI total score as the dependent variable. Given the positively skewed and strictly non‐negative distribution of PSQI scores, a gamma distribution with a log link function was applied. Clinically relevant demographic, clinical, psychological, and laboratory variables were simultaneously entered into the multivariable GLM. Multicollinearity was assessed using VIF values prior to model fitting. Results were presented as regression coefficients (*β*) with 95% Wald confidence intervals and corresponding *p* values.

To explore whether associations between inflammatory–nutritional markers and sleep quality differed according to depression status, stratified GLM analyses were performed based on Hospital Anxiety and Depression Scale–Depression (HADS‐D) categories (HADS‐D < 8 vs. ≥ 8). In these stratified models, HADS‐D total score was excluded from the multivariable analyses to avoid collinearity and overadjustment, as depression status served as the stratification variable. These analyses were considered exploratory.

All statistical tests were two‐sided, and *p* < 0.05 was considered statistically significant.

### 2.8 Ethics Approval

This study was approved by the Ethics Committee of İzmir Katip Çelebi University (Approval date: 15 January 2024; Approval number: 2805). The study was conducted in line with the principles of the Declaration of Helsinki. Written informed consent was obtained from all participants prior to inclusion in the study.

## 3 Results

### 3.1 Patient Characteristics and Distribution According to Sleep Quality

A total of 738 newly diagnosed cancer patients were included. Of these, 429 (58.1%) were classified as having PSQ (PSQI ≥ 5), and 309 (41.9%) were classified as having GSQ (PSQI < 5). The sociodemographic, clinical, and laboratory characteristics of the participants according to sleep quality are summarized in Table 1.

**TABLE 1:** Sociodemographic, clinical, and laboratory characteristics of participants stratified by sleep quality status (PSQI < 5 vs. ≥ 5).

Variables	Good sleep quality (PSQI < 5) (*n* = 309)	Poor sleep quality (PSQI ≥ 5) (*n* = 429)	Total (*N* = 738)	*p*
Age, *mean ±* SD	54.1 ± 12.4	56.0 ± 12.1	55.2 ± 12.2	**0.034**
Sex, *n* (%)				0.931
Male	115 (37.2)	161 (37.5)	276 (37.4)	
Female	194 (62.8)	268 (62.5)	462 (62.6)	
Education status, *n* (%)				**<** **0.001**
Illiterate	57 (18.4)	72 (16.8)	129 (17.5)	
Primary school	129 (41.7)	251 (58.5)	380 (51.5)	
High school	82 (26.5)	82 (19.1)	164 (22.2)	
University	41 (13.3)	24 (5.6)	65 (8.8)	
Monthly income, TL, *n* (%)				**<** **0.001**
Low	71 (23.0)	137 (31.9)	208 (28.2)	
Lower‐middle	138 (44.7)	208 (48.5)	346 (46.9)	
Upper‐middle	69 (22.3)	68 (15.9)	137 (18.6)	
High	31 (10.0)	16 (3.7)	47 (6.4)	
Place of residence, *n* (%)				0.903
Metropolitan	161 (52.1)	216 (50.3)	377 (51.1)	
Urban	21 (6.8)	30 (7.0)	51 (6.9)	
Semi‐urban	77 (24.9)	117 (27.3)	194 (26.3)	
Rural	50 (16.2)	66 (15.4)	116 (15.7)	
With children, *n* (%)	270 (87.4)	388 (90.4)	658 (89.2)	0.187
Number of children, *median* (IQR)	2 (1–3)	2 (2–3)	2 (1–3)	0.198
Employment status, *n* (%)				**<** **0.001**
Self‐employed	49 (15.9)	75 (17.5)	124 (16.8)	
Homemaker	118 (38.2)	209 (48.7)	327 (44.3)	
Civil servant	13 (4.2)	8 (1.9)	21 (2.8)	
Private sector	24 (7.8)	9 (2.1)	33 (4.5)	
Retired	105 (34.0)	128 (29.8)	233 (31.6)	
Cancer type, *n* (%)				0.192
Breast	122 (39.5)	160 (37.3)	282 (38.2)	
Lung	13 (4.2)	22 (5.1)	35 (4.7)	
Colorectal	55 (17.8)	80 (18.6)	135 (18.3)	
Prostate	10 (3.2)	14 (3.3)	24 (3.3)	
Stomach	21 (6.8)	24 (5.6)	45 (6.1)	
Pancreas	16 (5.2)	34 (7.9)	50 (6.8)	
Endometrium	9 (2.9)	19 (4.4)	28 (3.8)	
Ovary	6 (1.9)	21 (4.9)	27 (3.7)	
Renal	7 (2.3)	7 (1.6)	14 (1.9)	
Bladder	6 (1.9)	10 (2.3)	16 (2.2)	
Other	44 (14.3)	38 (8.8)	82 (11.1)	
Disease stage, *n* (%)				**<** **0.001**
Localized	206 (66.7)	143 (33.3)	349 (47.3)	
Locally advanced	14 (4.5)	14 (3.3)	28 (3.8)	
Metastatic	89 (28.8)	272 (63.4)	361 (48.9)	
Planned systemic anticancer treatment, *n* (%)	216 (69.9)	423 (98.6)	639 (86.6)	**<** **0.001**
LDH, U/L, *median* (IQR)	194 (160–231)	498 (393–711)	313 (205–532)	**<** **0.001**
Albumin, g/dL, *mean ±* SD	3.6 ± 0.5	2.8 ± 0.4	3.1 ± 0.4	**<** **0.001**
Neutrophils, 10^9^/L, *mean ±* SD	5.0 ± 0.8	9.5 ± 2.4	7.6 ± 2.1	**<** **0.001**
Lymphocytes, 10^9^/L, *mean ±* SD	1.7 ± 0.5	1.9 ± 0.8	1.8 ± 0.7	**<** **0.001**
Monocytes, 10^9^/L, *mean ±* SD	0.6 ± 0.2	0.6 ± 0.2	0.6 ± 0.2	**<** **0.001**
Platelets, 10^9^/L, *mean ±* SD	219 ± 67	305 ± 92	269 ± 85	**<** **0.001**
Inflammatory indices, *median* (IQR)				
NLR	3.1 (2.4–3.9)	5.1 (3.8–8.3)	4.1 (2.6–5.9)	**<** **0.001**
AISI	43 (31–54)	74 (36–220)	50 (33–92)	**<** **0.001**
SII	6.7 (5.2–7.9)	14.5 (7.8–30.7)	8.4 (5.9–16.8)	**<** **0.001**
SIRI	2.0 (1.4–2.5)	3.1 (1.5–6.2)	2.3 (1.4–3.4)	**<** **0.001**
LAR	56 (43–67)	166 (127–281)	114 (59–187)	**<** **0.001**
HADS‐D total score, *median* (IQR)	1 (0–2)	9 (7–11)	6 (1–10)	**<** **0.001**
HADS‐D categories, *n* (%)				**<** **0.001**
0–7	285 (92.2)	139 (32.4)	424 (57.5)	
8–10	16 (5.2)	169 (39.4)	185 (25.1)	
> 10	8 (2.6)	121 (28.2)	129 (17.5)	

*Note: p* values for categorical variables with more than two categories represent overall χ^2^ tests. Continuous variables were compared using the independent‐samples *t* test or Mann–Whitney *U* test, as appropriate. Bold values indicate statistically significant results (*p* < 0.05), consistent with the threshold specified in the Section 2.7.

Abbreviations: AISI, aggregate index of systemic inflammation; HADS‐D, Hospital Anxiety and Depression Scale–Depression subscale; IQR, interquartile range; LAR, lactate dehydrogenase‐to‐albumin ratio; LDH, lactate dehydrogenase; NLR, neutrophil‐to‐lymphocyte ratio; PSQI, Pittsburgh Sleep Quality Index; SD, standard deviation; SII, systemic immune‐inflammation index; SIRI, systemic inflammation response index.

Patients with PSQ were older than those with GSQ (mean age: 56.0 ± 12.1 vs. 54.1 ± 12.4 years; *p* = 0.034). Although no significant difference was observed in sex distribution between the groups, educational level and monthly income differed significantly by sleep quality status.

The overall distribution of cancer types did not differ significantly between groups (*p* = 0.192). Disease stage, however, differed significantly between groups; patients with PSQ had a markedly higher prevalence of metastatic disease (*p* < 0.001). Similarly, the proportion of patients with planned systemic anticancer treatment was significantly higher in the PSQ group (*p* < 0.001).

Regarding laboratory parameters, patients with PSQ exhibited higher lactate dehydrogenase (LDH) levels and lower albumin levels compared to those with GSQ (both *p* < 0.001). In terms of complete blood count parameters, neutrophil and platelet counts were higher in the PSQ group, while lymphocyte and monocyte counts differed significantly between groups (all *p* < 0.001).

All systemic inflammatory indices were significantly elevated in patients with PSQ. NLR, aggregate index of systemic inflammation (AISI), SII, SIRI, and LAR levels were all markedly higher compared to those in the GSQ group (all comparisons *p* < 0.001).

With respect to depressive symptoms, HADS‐D total scores were significantly higher in the PSQ group [median (IQR): 9 [7, 8, 9, 10, 11] versus 1 (0–2); *p* < 0.001]. Moreover, categorical analysis based on HADS‐D thresholds showed clinically significant depressive symptoms (HADS‐D ≥ 8) to be more prevalent among patients with PSQ (*p* < 0.001).

### 3.2 Factors Associated With Poor Sleep Quality: Logistic Regression Analysis

Univariable and multivariable logistic regression analyses were performed to identify factors associated with PSQ (defined as PSQI ≥ 5) (Table 2).

**TABLE 2:** Binary logistic regression analysis of factors associated with poor sleep quality (PSQI ≥ 5).

Variables	Univariate			Multivariate		
	OR	95% CI	*p*	OR	95% CI	*p*
Age, *(per a year increase)*	1.013	1.001–1.025	**0.034**	1.016	0.989–1.045	0.248
Female sex *(vs. male)*	0.987	0.729–1.335	0.931	0.388	0.161–0.934	**0.035**
Residence				—	—	—
Metropol *(ref)*	—	Reference	—			
Urban	1.065	0.588–1.928	0.836			
Semi‐urban	1.133	0.796–1.611	0.489			
Rural	0.984	0.646–1.498	0.940			
Monthly income						
Low *(ref)*	—	Reference	—	—	Reference	—
Lower‐middle	0.781	0.546–1.118	0.177	0.820	0.393–1.712	0.598
Upper‐middle	0.511	0.329–0.794	**0.003**	0.929	0.358–2.413	0.880
High	0.267	0.137–0.522	**< 0.001**	0.364	0.084–1.570	0.175
Education status						
Illiterate *(ref)*	—	Reference	—	—	Reference	—
Primary school	1.540	1.025–2.315	**0.038**	3.589	1.446–8.906	**0.006**
High school	0.792	0.498–1.258	0.323	2.801	0.985–7.963	0.053
University	0.463	0.251–0.855	**0.014**	1.470	0.321–6.733	0.620
Employment status						
Retired *(ref)*	—	Reference	—	—	Reference	—
Homemaker	1.453	1.031–2.047	**0.033**	2.382	0.904–6.280	0.079
Civil servant	0.505	0.202–1.264	0.144	0.414	0.077–2.233	0.305
Private sector	0.308	0.137–0.690	**0.004**	1.561	0.328–7.439	0.576
Self‐employed	1.256	0.806–1.955	0.314	1.164	0.454–2.981	0.752
With children, *(yes)*	1.367	0.859–2.176	0.188	—	—	—
Number of children, *(per one point increase)*	1.042	0.938–1.158	0.441	—	—	—
Disease stage						
Localized *(ref)*	—	Reference	—	—	Reference	—
Locally advanced	1.441	0.666–3.114	0.353	1.031	0.272–3.907	0.964
Metastatic	4.403	3.195–6.067	**< 0.001**	1.730	0.964–3.107	0.066
Planned systemic anticancer treatment, *(yes)*	—	N/A^a^	—	—	—	—
HADS‐D total score, *(per one point increase)*	1.924	1.765–2.097	**< 0.001**	1.913	1.732–2.113	**< 0.001**
NLR, *(per one point increase)*	1.687	1.521–1.871	**< 0.001**	1.858	1.474–2.343	**< 0.001**
AISI, *(per one point increase)*	1.022	1.016–1.027	**< 0.001**	—	—	—
SII, *(per one point increase)*	1.306	1.239–1.376	**< 0.001**	—	—	—
SIRI, *(per one point increase)*	1.644	1.474–1.833	**< 0.001**	—	—	—
LAR, *(per one point increase)*	1.294	1.189–1.409	**< 0.001**	—	—	—

*Note:* Model performance: Omnibus *χ* ^2^ = 655.1, df = 16, *p* < 0.001; Nagelkerke *R* ^2^ = 0.792. Model estimates were internally validated using bootstrap resampling (1000 iterations). Hosmer–Lemeshow statistics are reported for completeness, but should be interpreted cautiously because of their known sensitivity to large sample sizes. Bold values indicate statistically significant results (*p* < 0.05), consistent with the threshold specified in the Section 2.7.

Abbreviations: AISI, aggregate index of systemic inflammation; CI, confidence interval; HADS‐D, Hospital Anxiety and Depression Scale–Depression subscale; LAR, lactate dehydrogenase‐to‐albumin ratio; NLR, neutrophil‐to‐lymphocyte ratio; OR, odds ratio; PSQI, Pittsburgh Sleep Quality Index; SII, systemic immune‐inflammation index; SIRI, systemic inflammation response index.

^a^ Planned systemic anticancer treatment was not entered into the multivariable model because of near‐complete separation by sleep quality group (98.6% vs. 69.9%; see Table 1), which precluded stable coefficient estimation.

In univariable analyses, older age, metastatic disease stage, depressive symptom severity (HADS‐D), NLR, AISI, SII, SIRI, and LAR were all significantly associated with PSQ (all *p* < 0.001); whereas sex, place of residence, having children, and number of children were not.

Variables with *p* < 0.10 in univariable analyses, together with clinically relevant covariates, were entered into the multivariable logistic regression model. In the multivariable analysis, depressive symptom severity (HADS‐D total score) remained independently and strongly associated with PSQ (odds ratio [OR] = 1.913; 95% CI: 1.732–2.113; *p* < 0.001). In addition, NLR was independently associated with PSQ after adjustment for other covariates (OR = 1.858; 95% CI: 1.474–2.343; *p* < 0.001).

In the multivariable model, age and disease stage lost statistical significance, whereas female sex was independently associated with a lower likelihood of PSQ (OR = 0.388; 95% CI: 0.161–0.934; *p* = 0.035). Sociodemographic variables, including educational level, monthly income, and employment status, did not show independent associations with PSQ.

Regarding model performance, the multivariable logistic regression model was statistically significant overall (Omnibus *χ* ^2^ = 655.1; *p* < 0.001) and demonstrated high explanatory power, with a Nagelkerke *R* ^2^ value of 0.792.

Internal validation of the multivariable model using bootstrap resampling (1000 iterations) demonstrated stable parameter estimates for the main predictors. The association between depressive symptom severity and PSQ remained significant, with bootstrap bias‐corrected and accelerated (BCa) 95% CI for the regression coefficient ranging from 0.516 to 0.946. Similarly, the association for NLR remained consistent (bootstrap BCa 95% CI for the regression coefficient: 0.409–1.013), supporting the robustness of the model estimates.

### 3.3 Factors Associated With Total PSQI Score: Generalized Linear Model Analysis

Sleep disturbance severity was analyzed using univariable and multivariable generalized linear models (GLMs) with PSQI score as a continuous outcome (Table 3). Because PSQI scores showed a positively skewed distribution, a gamma distribution with a log link was applied.

**TABLE 3:** Univariable and multivariable generalized linear model (GLM) analyses of factors associated with total PSQI score.

	Univariate			Multivariate		
Variables	*β*	95% CI	*p*	*β*	95% CI	*p*
Age	0.003	−0.001–0.008	0.150	0.005	0.002–0.008	0.001
Female sex	0.039	−0.072–0.151	0.490	0.032	−0.063–0.127	0.511
Residence				—	—	—
Metropolitan (ref)	—	Reference	—			
Urban	0.013	−0.143–0.168	0.871			
Semi‐urban	−0.009	−0.255–0.237	0.940			
Rural	0.018	−0.154–0.190	0.838			
Monthly income						
High *(ref)*	—	Reference	—	—	Reference	—
Low	0.406	0.172–0.641	**0.001**	0.140	−0.014–0.295	0.075
Lower‐middle	0.342	0.116–0.567	**0.003**	0.092	−0.052–0.235	0.210
Upper‐middle	0.190	−0.056–0.435	0.129	0.115	−0.031–0.261	0.122
Education status						
University (ref)	—	Reference	—	—	Reference	—
Illiterate	0.232	0.011–0.453	0.040	0.018	−0.141–0.177	0.822
Primary school	0.288	0.093–0.483	0.004	0.127	−0.014–0.269	0.078
High school	0.113	−0.101–0.326	0.301	0.130	−0.008–0.267	0.064
Employment status						
Homemaker *(ref)*	—	Reference	—	—	Reference	—
Retired	−0.092	−0.217–0.033	0.148	−0.082	−0.188–0.025	0.134
Civil servant	−0.233	−0.561–0.094	0.163	0.149	−0.064–0.362	0.169
Private sector	−0.354	−0.620 to −0.088	**0.009**	0.167	−0.009–0.342	0.062
Self‐employed	−0.073	−0.277–0.081	0.351	0.013	−0.104–0.130	0.825
With children	0.051	−0.122–0.255	0.562	—	—	—
Number of children	0.013	−0.027–0.053	0.526	—	—	—
Disease stage						
Locally advanced *(ref)*	—	Reference	—	—	Reference	—
Localized	−0.090	−0.363–0.184	0.519	−0.051	−0.216–0.114	0.543
Metastatic	0.416	0.143–0.689	**0.003**	0.104	−0.063–0.270	0.222
HADS‐D total score	0.143	0.136–0.151	**<** **0.001**	0.128	0.118–0.138	**<** **0.001**
NLR	0.067	0.056–0.077	**<** **0.001**	—	—	—
AISI	0.003	0.003–0.004	**<** **0.001**	—	—	—
SII	0.017	0.014–0.019	**<** **0.001**	—	—	—
SIRI	0.122	0.103–0.141	**<** **0.001**	—	—	—
LAR score (10×)	0.033	0.029–0.037	**<** **0.001**	0.005	0.002–0.008	**0.001**

*Note:* *Model performance:* Omnibus likelihood ratio test: *χ* ^2^ = 918.6, df = 16, *p* < 0.001. *Reference categories:* male for sex, metropolitan residence, high income for income level, university education level, homemaker for occupation, and locally advanced stage for cancer stage. *Model specification:* Given the strong correlation between lactate dehydrogenase–to–albumin ratio (LAR) and neutrophil‐to‐lymphocyte ratio (NLR) (*r* = 0.70), inflammatory indices were not included simultaneously in the same multivariable model to avoid multicollinearity. LAR was selected for the generalized linear model to better reflect the combined inflammatory–nutritional burden associated with the continuous PSQI total score. LAR was scaled by a factor of 10 in regression analyses to improve interpretability of regression coefficients. Bold values indicate statistically significant results (*p* < 0.05), consistent with the threshold specified in the Section 2.7.

Abbreviations: AISI, aggregate index of systemic inflammation; CI, confidence interval; HADS‐D, Hospital Anxiety and Depression Scale–Depression subscale; IQR, interquartile range; LAR, lactate dehydrogenase–to–albumin ratio; N/A, not applicable; NLR, neutrophil‐to‐lymphocyte ratio; PSQI, Pittsburgh Sleep Quality Index; ref, reference; SII, systemic immune‐inflammation index; SIRI, systemic inflammation response index.

Given the positively skewed distribution of PSQI scores, a generalized linear model with a gamma distribution and log link function was applied. In univariable analyses, depressive symptom severity measured by HADS‐D, systemic inflammatory indices (NLR, AISI, SII, SIRI, and LAR), and metastatic disease stage were significantly associated with total PSQI score (all *p* < 0.001). Age, sex, place of residence, having children, and number of children were not significantly associated with PSQI score.

Variables with *p* < 0.10 in univariable analyses and clinically relevant covariates were included in the multivariable model. Depressive symptom severity remained independently and strongly associated with higher PSQI scores (*β* = 0.128; 95% CI 0.118–0.138; *p* < 0.001). LAR was also independently associated with PSQI score after adjustment (*β* = 0.005; 95% CI 0.002–0.008; *p* = 0.001). Age showed a modest independent association (*β* = 0.005; 95% CI 0.002–0.008; *p* = 0.001), whereas sex, educational level, monthly income, occupational status, and disease stage were not significant in the multivariable model.

Overall model fit was significant (Omnibus likelihood ratio test *χ* ^2^ = 918.6, df = 16, *p* < 0.001). In sensitivity analyses using an alternative gamma GLM specification, HADS‐D score and LAR remained independently associated with PSQI score (Table S1), supporting the robustness of the findings.

### 3.4 Discrimination of Poor Sleep Quality: ROC Analyses

**FIGURE 1:**
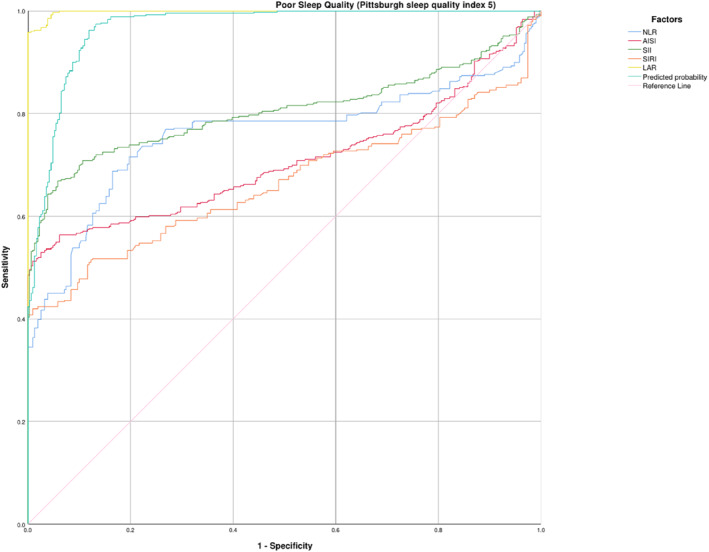
ROC curves illustrating the discriminatory performance of depressive symptom burden and systemic inflammatory indices for PSQ, defined as a PSQI total score ≥ 5. ROC curves are shown for HADS‐D, NLR, AISI, SII, SIRI, and LAR. Diagonal line represents the line of no discrimination. Corresponding AUC values, 95% CI, and optimal cut‐off points are presented in Table 4.

ROC analyses were performed to evaluate the ability of depressive symptom severity and systemic inflammatory indices to discriminate PSQ (PSQI ≥ 5) (Table 4, Figure 1).

**TABLE 4:** Receiver operating characteristic (ROC) analysis of depressive symptoms and inflammatory indices for the discrimination of poor sleep quality (PSQI ≥ 5).

	Poor sleep quality			Sensitivity (%)	Specificity (%)
Variables	AUC (95% CI)	*p*	Cut‐off^b^		
HADS‐D total score	0.941 (0.922–0.961)	**<** **0.001**	≥ 3.5	99	84
NLR	0.759 (0.724–0.795)	**<** **0.001**	≥ 3.97	74	78
AISI	0.708 (0.670–0.746)	**<** **0.001**	≥ 73.0	51	99
SII	0.805 (0.772–0.837)	**<** **0.001**	≥ 10.3	67	94
SIRI	0.664 (0.625–0.704)	**<** **0.001**	≥ 1.27	79	18
LAR	0.998 (0.997–0.999)	**<** **0.001**	≥ 99.7	96	99
Predictive values of multivariate regression model in Table 2a	0.967 (0.955–0.979)	**<** **0.001**	≥ 0.3	96	88

*Note:* Bold values indicate statistically significant results (*p* < 0.05), consistent with the threshold specified in the Section 2.7.

Abbreviations: AISI, aggregate index of systemic inflammation; AUC, area under the curve; CI, confidence interval; HADS‐D, Hospital Anxiety and Depression Scale–Depression subscale; LAR, lactate dehydrogenase‐to‐albumin ratio; NLR, neutrophil‐to‐lymphocyte ratio; PSQI, Pittsburgh Sleep Quality Index; SII, systemic immune‐inflammation index; SIRI, systemic inflammation response index.

^a^ Predicted probabilities derived from the multivariable logistic regression model presented in Table 2 were used to construct the ROC curve.

^b^ Cut‐off values were determined using the Youden index and are intended for statistical discrimination rather than validated clinical decision‐making.

Among all variables HADS‐D demonstrated the strongest discriminative performance (AUC = 0.941; 95% CI 0.922–0.961; *p* < 0.001). Using the Youden index, the optimal cut‐off value was ≥ 3.5, corresponding to a sensitivity of 99% and specificity of 84%.

Among inflammatory markers, NLR showed moderate‐to‐good discriminative ability (AUC = 0.759; 95% CI 0.724–0.795; *p* < 0.001), with an optimal cut‐off of ≥ 3.97 (sensitivity 74%, specificity 78%). SII and AISI also demonstrated significant discrimination with AUC values of 0.805 (95% CI: 0.772–0.837) and 0.708 (95% CI: 0.670–0.746), respectively (both *p* < 0.001), whereas SIRI showed more limited performance (AUC = 0.664; 95% CI 0.625–0.704; *p* < 0.001).

Notably, LAR showed very high apparent discrimination (AUC = 0.998; 95% CI 0.997–0.999; *p* < 0.001), with an optimal cut‐off of ≥ 99.7 (sensitivity 96%, specificity 99%). This remarkably high discriminative capacity should be interpreted cautiously and may reflect the ability of LAR to capture both systemic inflammatory activity and nutritional–metabolic status, thereby providing a more integrative representation of the biological burden associated with sleep disturbance.

Sensitivity analyses were conducted to assess the potential influence of extreme values on the near‐perfect discriminative ability of LAR. ROC analyses using log‐transformed LAR—to reduce skewness—yielded similar results (AUC = 0.998; 95% CI 0.997–1.000). After exclusion of potential outliers identified from logistic regression diagnostics (standardized residuals > 3 or extreme predicted probabilities), the discriminative performance remained high (AUC = 0.994; 95% CI 0.990–0.998; *n* = 373), supporting the robustness of the findings.

Further, a ROC curve constructed using the predicted probabilities derived from the multivariable logistic regression model demonstrated excellent discriminative performance for identifying PSQ (AUC = 0.967; 95% CI: 0.955–0.979; *p* < 0.001).

The exceptionally high discriminative performance observed for LAR should be interpreted with caution. Given that LAR integrates both inflammatory activity and nutritional–metabolic status, its strong association with sleep disturbance may partly reflect overlapping systemic disease burden at diagnosis rather than a standalone diagnostic marker. External validation in independent cohorts is therefore warranted.

In addition, a ROC curve using the predicted probabilities derived from the multivariable logistic regression model demonstrated excellent discriminative performance for identifying PSQ (AUC = 0.967; 95% CI: 0.955–0.979; *p* < 0.001).

The exceptionally high discriminative performance observed for LAR should be interpreted with caution. Given that LAR integrates both inflammatory activity and nutritional–metabolic status, its strong association with sleep disturbance may partly reflect overlapping systemic disease burden at diagnosis rather than a standalone diagnostic marker. External validation in independent cohorts is therefore warranted.

### 3.5 Stratified Generalized Linear Model Analyses According to Depression Status

Generalized linear models stratified by depression status (HADS‐D < 8 vs. ≥ 8) assessed the association between systemic inflammatory–nutritional burden and sleep disturbance severity (PSQI total score) (Table 5).

**TABLE 5:** Multivariable generalized linear models (GLMs) examining factors associated with total PSQI score, stratified by depression status (HADS‐D < 8 vs. ≥ 8).

Variables	No depression (HADS‐D < 8; *N* = 424)			Depression (HADS‐D ≥ 8; *N* = 314)		
	*β*	95% CI	*p*	*β*	95% CI	*p*
Age	0.004	−0.001–0.009	0.054	−0.002	−0.004–0.001	0.105
Female sex	−0.014	−0.147–0.119	0.840	−0.011	−0.088–0.066	0.778
Monthly income						
High *(ref)*	—	Reference	—	—	Reference	—
Low	−0.014	−0.231–0.204	0.903	0.030	−0.101–0.161	0.653
Lower‐middle	−0.013	−0.212–0.185	0.894	0.043	−0.082–0.168	0.500
Upper‐middle	0.039	−0.160–0.239	0.698	−0.023	−0.152–0.105	0.723
Education status						
University *(ref)*	—	Reference	—	—	Reference	—
Illiterate	0.280	0.053–0.506	**0.016**	−0.011	−0.142–0.121	0.875
Primary school	0.325	0.131–0.519	**0.001**	−0.030	−0.152–0.091	0.627
High school	0.290	0.104–0.477	**0.002**	−0.037	−0.158–0.084	0.549
Employment status						
Homemaker *(ref)*	—	Reference	—	—	Reference	—
Retired	−0.037	−0.190–0.115	0.634	−0.017	−0.099–0.066	0.691
Civil servant	0.261	−0.022–0.543	0.070	0.030	−0.162–0.222	0.759
Private sector	0.282	0.051–0.513	**0.017**	−0.063	−0.229–0.103	0.459
Self‐employed	−0.039	−0.208–0.130	0.652	−0.003	−0.093–0.087	0.951
Disease stage						
Locally advanced *(ref)*	—	Reference	—	—	Reference	—
Localized	−0.021	−0.254–0.213	0.863	0.012	−0.121–0.144	0.864
Metastatic	0.271	0.030–0.511	**0.027**	0.057	−0.075–0.188	0.398
LAR score (10×)	0.107	0.097–0.117	**<** **0.001**	0.031	0.009–0.051	**<** **0.001**

*Note:* Generalized linear models with a gamma distribution and log link were fitted separately for patients without depression (HADS‐D < 8) and with depression (HADS‐D ≥ 8). Reference categories were male for sex, high income for monthly income, university education, homemaker for employment status, and locally advanced disease for disease stage. LAR was scaled by a factor of 10 to improve interpretability of the regression coefficients. Bold values indicate statistically significant results (*p* < 0.05), consistent with the threshold specified in the Section 2.7.

Abbreviations: CI, confidence interval; HADS‐D, Hospital Anxiety and Depression Scale–Depression subscale; LAR, lactate dehydrogenase‐to‐albumin ratio; PSQI, Pittsburgh Sleep Quality Index; ref, reference.

Among patients without clinically significant depressive symptoms (HADS‐D < 8; *n* = 424), LAR was independently associated with PSQI score after adjustment for demographic, socioeconomic, and disease‐related variables (*β* = 0.107; 95% CI: 0.097–0.117; *p* < 0.001). Age, sex, socioeconomic variables, and disease stage were not independently associated with total PSQI score. Similarly, among patients with clinically significant depressive symptoms (HADS‐D ≥ 8; *n* = 314), LAR remained independently associated with PSQI score (*β* = 0.031; 95% CI: 0.009–0.051; *p* < 0.001), while age, sex, education, income, occupation, and disease stage were not significantly associated with total PSQI score.

The persistence of an independent association between LAR and total PSQI score across both depression strata indicates that the relationship between sleep disturbance severity and systemic inflammatory–nutritional burden is maintained irrespective of depressive symptom status.

Notably, the magnitude of the association between LAR and sleep disturbance severity appeared greater among patients without depressive symptoms, suggesting that inflammatory–nutritional burden may play a more prominent role in shaping sleep disturbance when depressive symptomatology is absent.

## 4 Discussion

In our study, sleep disturbance was highly prevalent among newly diagnosed cancer patients prior to treatment initiation and independently associated with both depressive symptoms and systemic inflammatory burden. Evaluating sleep using both a dichotomous indicator of PSQ (PSQI ≥ 5) and continuous PSQI scores suggests that sleep disturbance at diagnosis reflects not only psychological distress but also a broader biobehavioral process involving biological and emotional factors. These findings suggest that sleep disturbance at diagnosis may serve as a clinically relevant indicator associated with both systemic disease burden and psycho‐oncological vulnerability.

Evaluations conducted later in the treatment trajectory are inevitably influenced by substantial clinical heterogeneity, including variations in treatment modalities (e.g., chemotherapy, immunotherapy, hormone therapy, targeted agents), treatment duration, cumulative toxicities, treatment response, disease progression, and exposure to medications such as corticosteroids, which may influence sleep regulation. These evolving and treatment‐specific factors are known to directly affect sleep regulation and may introduce additional sources of variability that complicate interpretation. By examining patients prior to treatment initiation, we sought to characterize sleep disturbance within a comparatively less confounded biological and psychological context. Nevertheless, the decision to focus specifically on the time of diagnosis warrants further clarification.

The strong association between depressive symptoms and sleep disturbance is well established in the psycho‐oncology literature, and our findings are consistent with this existing body of evidence. The frequent co‐occurrence of depression and sleep disturbance among cancer patients has been attributed to the psychological burden arising from emotional distress, uncertainty, and illness perception at the time of diagnosis [1, 3, 6, 11]. In our study, the independent association of the HADS‐D total score with both the presence of PSQ and the severity of sleep disturbance supports the notion that depressive symptoms constitute one of the key determinants of sleep regulation in this early phase. However, depression alone did not fully explain sleep disturbance. Systemic inflammatory indices remained independently associated with sleep outcomes even after adjustment for depressive symptoms. These findings support emerging psycho‐oncology frameworks suggesting that sleep disturbance should not be viewed solely as a secondary manifestation of depression but rather as a partially autonomous biobehavioral process [1, 8, 20].

In this context, the relationship between depression and sleep disturbance appears to be bidirectional rather than unidirectional, exhibiting a reciprocal and multilayered structure. While depressive symptoms may exacerbate sleep disturbance, disrupted sleep patterns have also been proposed to negatively affect emotional regulation, thereby perpetuating depressive symptoms [1, 11]. However, our findings indicate that biological components—particularly systemic inflammatory and nutritional burden—make a meaningful contribution to this cycle, underscoring that sleep disturbance observed at the time of cancer diagnosis should be evaluated beyond purely psychological factors.

One of the most notable findings of our study is that systemic inflammatory burden was associated with sleep disturbance independently of depressive symptoms. The demonstration of independent contributions of inflammatory indices in both logistic regression analyses assessing the presence of PSQ and generalized linear models examining sleep disturbance severity as a continuous PSQI score provides strong support for the biological components underlying sleep disturbance. In this context, ROC analyses demonstrate that both depressive symptom burden and systemic inflammatory burden jointly contribute to the clinical discrimination of PSQ. The concordance between these findings supports the interpretation that both psychological and biological factors contribute to the observed associations.

Importantly, the high explanatory and discriminative performance observed in multivariable models was to a substantial extent driven by depressive symptom burden, which is consistent with the well‐established link between depression and sleep disturbance. However, the persistence of independent associations between inflammatory–nutritional indices and sleep disturbance across complementary analytical approaches indicates that biological vulnerability contributes beyond psychological distress alone.

In particular, the observation that readily accessible inflammatory indices such as NLR and LAR demonstrated significance across different analytical contexts sheds light on the heterogeneous nature of sleep disturbance. The prominence of NLR in distinguishing the presence of clinically significant sleep disturbance may be related to its sensitivity in reflecting acute systemic inflammatory responses. In contrast, the association between LAR—which integratively captures both inflammatory and nutritional status—and sleep disturbance severity suggests that sleep regulation is shaped not only by inflammation, but also by a broader biological substrate influenced by metabolic and catabolic burden [19, 20]. Taken together, these findings support the notion that distinct inflammatory pathways may differentially shape the presence versus the severity of sleep disturbance at diagnosis. It is also possible that the strong discriminative performance of LAR partly reflects its close association with overall disease burden and nutritional status at diagnosis, which may themselves influence sleep regulation.

This biological framework is also consistent with psychoneuroimmunological models. It is well established that sleep disturbance can increase the release of proinflammatory cytokines, while inflammatory signals may, in turn, influence sleep regulation, energy balance, and emotional regulation through central nervous system pathways [1, 8, 11]. In the context of cancer, the amplification of these processes suggests that tumor‐related inflammation and host immune responses may play a direct role in the development of sleep disturbance.

Our stratified analyses demonstrated that this biological association persisted independently of the presence of depressive symptoms. The finding that LAR remained strongly associated with sleep disturbance severity even among patients without depression suggests that, in a subset of patients, sleep disturbance may primarily represent a vulnerability marker shaped by inflammatory–nutritional burden. This observation is consistent with emerging psycho‐oncology literature proposing that depression and related symptoms may exhibit distinct biological subtypes, and that inflammation‐based phenotypes may be clinically distinguishable [7, 20, 21]. Accordingly, LAR was not conceptualized as a standalone predictive marker but rather as an integrative indicator of systemic inflammatory–nutritional burden.

Importantly, the persistence of this association even among patients without clinically significant depressive symptoms suggests that sleep disturbance at the time of cancer diagnosis may reflect a broader biobehavioral phenotype rather than merely a manifestation of psychological distress. This observation supports the interpretation that inflammatory–nutritional burden may constitute an independent biological substrate contributing to sleep disturbance during the diagnostic phase.

The stronger association observed between inflammatory–nutritional burden and sleep disturbance severity among patients without depressive symptoms further suggests a potential effect modification by depression status, whereby biological mechanisms may play a more prominent role in shaping sleep disturbance in the absence of overt affective symptomatology.

Although LAR demonstrated an exceptionally high discriminative capacity for poor sleep quality, this finding should not be interpreted as suggesting a diagnostic role for LAR in isolation. Rather, it underscores the extent to which sleep disturbance at cancer diagnosis may mirror global inflammatory–nutritional burden. The magnitude of discrimination observed in the present cohort highlights biological coupling at diagnosis and calls for cautious interpretation and external validation. Accordingly, these findings should be viewed as hypothesis‐generating rather than definitive, particularly with regard to clinical prediction.

### 4.1 Clinical Implications

From a clinical perspective, the association between sleep disturbance and routinely available inflammatory indices may have practical implications. Simple biomarkers such as NLR and LAR may complement patient‐reported assessments and help identify biologically vulnerable patients at the time of diagnosis, including those who do not exhibit prominent depressive symptoms.

Routine screening for sleep disturbance at diagnosis, together with awareness of inflammatory–nutritional burden, may facilitate early identification of patients who could benefit from supportive interventions. Integrating sleep assessment into psycho‐oncological evaluation may therefore help guide timely referral to supportive care services.

These findings also suggest that interventions for sleep disturbance should not be limited to psychological approaches alone. In patients with elevated inflammatory or nutritional burden, supportive strategies may require a broader framework that includes behavioral interventions, sleep hygiene, and management of underlying biological vulnerability.

### 4.2 Strengths and Limitations of the Study

A large sample size and the inclusion of exclusively newly diagnosed cancer patients before any oncological treatment are the strengths of our study. This design minimized potential biological and psychological confounding effects related to treatment and allowed for a clearer assessment of the natural biobehavioral profile of sleep disturbance at the time of diagnosis. In addition, analyzing sleep quality using both dichotomous and continuous outcomes, together with the application of complementary statistical approaches, further enhanced the methodological robustness of the study.

Several limitations of this study should be acknowledged. Firstly, the cross‐sectional design precludes causal inferences and limits conclusions regarding temporal relationships among sleep disturbance, depressive symptoms, and inflammatory processes. Longitudinal studies are required to determine whether sleep disturbance observed at diagnosis predicts persistent symptom trajectories or subsequent clinical outcomes. Secondly, sleep quality was assessed using a self‐report questionnaire, and objective sleep measurements were not included. Thirdly, systemic inflammation was evaluated using indirect inflammatory indices rather than specific cytokine measurements, and the single‐center design may limit the generalizability of the findings. Moreover, the high discriminative performance observed for certain inflammatory indices may partly reflect shared variance with overall disease burden and should therefore be interpreted cautiously. Finally, the absence of external validation limits the generalizability of the proposed inflammatory–sleep associations, underscoring the need for replication in independent cohorts.

An additional limitation concerns the clinical applicability of the composite inflammatory indices evaluated in this study. Unlike established biomarkers with validated clinical thresholds, indices such as NLR and LAR currently lack universally accepted cut‐off values for routine clinical practice in psycho‐oncology. Accordingly, the cut‐off values identified in the present study were derived empirically from this cohort and should be regarded as exploratory rather than suitable for routine clinical implementation until standardized thresholds have been established and validated across independent populations. In addition, interpretation of these indices may not be straightforward for psychosocial oncology clinicians who are less familiar with inflammatory biomarker assessment, particularly given the influence of potential biological confounders such as occult infection, corticosteroid exposure, hydration status, nutritional status, and other cancer‐related systemic conditions. Nevertheless, because these indices are inexpensive and readily available from routine laboratory testing, they may ultimately serve as complementary biomarkers to support multidisciplinary risk stratification and the early identification of patients who could benefit from comprehensive psycho‐oncological assessment and supportive care, provided that practical guidance for their interpretation and clinically validated thresholds become available.

## 5 Conclusion

This study demonstrates that sleep disturbance is highly prevalent at the time of cancer diagnosis and closely associated not only with depressive symptoms but also with systemic inflammatory and nutritional burden. Our findings support the interpretation that sleep disturbance at diagnosis should not be viewed solely as a byproduct of depressive symptoms but may instead represent an early clinical signal shaped by the interplay between biological and psychological processes. Early identification of sleep disturbance through a comprehensive psycho‐oncological assessment at diagnosis may facilitate the recognition of vulnerable patient subgroups and enable the development of more targeted supportive care strategies.

## Author Contributions

**Zeynep Gülsüm Güç:** conceptualization, methodology, formal analysis, investigation, writing – original draft. **Hüseyin Döngelli:** formal analysis. **Hasan Güç:** methodology, formal analysis, investigation. **Ahmet Alacacıoğlu:** conceptualization, writing – review and editing, supervision. **Mustafa Oktay Tarhan:** conceptualization, writing – review and editing, supervision.

## Funding

The authors have nothing to report.

## Ethics Statement

The study was conducted in accordance with the principles of the Declaration of Helsinki and approved by the Ethics Committee of İzmir Katip Çelebi University Atatürk Training and Research Hospital (approval number: [2805], approval date: [15 January 2024]).

## Consent

Written informed consent was obtained from all participants included in the study.

## Conflicts of Interest

The authors declare no conflicts of interest.

## Supporting information


**Figure S1:** Relationship between lactate dehydrogenase–to–albumin ratio (LAR) and Pittsburgh Sleep Quality Index (PSQI) scores.

**Table S1:** Multivariable generalized linear model (gamma distribution with log link) examining factors associated with total PSQI score.

## Data Availability

The data supporting the findings of this study are available from the corresponding author upon reasonable request.
