# Supplementary material for: Sleep Disturbance at Cancer Diagnosis and Its Associations With Depressive Symptoms and Systemic Inflammatory Burden
Source: Psychooncology. 2026 Aug 11;35(8):e70576. doi: 10.1002/pon.70576 (PMC13459145; doi:10.1002/pon.70576)
Supplement: Supplementary file 1 [file PON-35-e70576-s001.docx]

**Supplementary Table 1.** Multivariable generalized linear model (gamma distribution with log link) examining factors associated with total PSQI score

| **Variable** | **B (β)** | **SE** | **Exp(B) (RR)** | **95% CI for Exp(B)** | **p-value** |
| --- | --- | --- | --- | --- | --- |
| **Age (years)** | 0.005 | 0.001 | 1.005 | 1.002 – 1.008 | **0.001** |
| **Sex (Female vs Male)** | 0.032 | 0.049 | 1.033 | 0.939 – 1.135 | 0.511 |
| **Education status** |  |  |  |  |  |
| Primary school | 0.127 | 0.072 | 1.135 | 0.986 – 1.309 | 0.078 |
| High school | 0.130 | 0.070 | 1.139 | 0.992 – 1.306 | 0.064 |
| **Monthly income level** |  |  |  |  |  |
| Low | 0.140 | 0.079 | 1.150 | 0.986 – 1.343 | 0.075 |
| Lower-middle | 0.092 | 0.073 | 1.096 | 0.949 – 1.264 | 0.210 |
| Upper-middle | 0.115 | 0.074 | 1.122 | 0.970 – 1.299 | 0.122 |
| **Employment status** |  |  |  |  |  |
| Retired | −0.082 | 0.054 | 0.921 | 0.829 – 1.026 | 0.134 |
| Civil servant | 0.149 | 0.109 | 1.161 | 0.938 – 1.437 | 0.169 |
| Private sector | 0.167 | 0.089 | 1.182 | 0.991 – 1.408 | 0.062 |
| Self-employed | 0.013 | 0.060 | 1.013 | 0.901 – 1.139 | 0.825 |
| **Disease stage** |  |  |  |  |  |
| Localized | −0.051 | 0.084 | 0.950 | 0.806 – 1.120 | 0.543 |
| Metastatic | 0.104 | 0.085 | 1.110 | 0.939 – 1.310 | 0.222 |
| **HADS score** | 0.128 | 0.005 | **1.137** | **1.125 – 1.148** | **<0.001** |
| **LAR score (×10)** | 0.005 | 0.001 | **1.005** | **1.002 – 1.008** | **0.001** |

**Abbreviations:** PSQI, Pittsburgh Sleep Quality Index; GLM, generalized linear model; HADS-D, Hospital Anxiety and Depression Scale–Depression subscale; LAR, lactate dehydrogenase–to–albumin ratio; RR, rate ratio; CI, confidence interval.

Reference categories were male for sex, high income for monthly income, university education, homemaker for employment status, and locally advanced disease for disease stage.

Regression coefficients represent comparisons with the respective reference categories. A generalized linear model with a gamma distribution and log link was fitted.

LAR was scaled by a factor of 10 to improve interpretability of regression coefficients.
