# Supplementary material for: Sleep Disturbance at Cancer Diagnosis and Its Associations With Depressive Symptoms and Systemic Inflammatory Burden
Source: Psychooncology. 2026 Aug 11;35(8):e70576. doi: 10.1002/pon.70576 (PMC13459145; doi:10.1002/pon.70576)
Supplement: Supplementary file 2 [file PON-35-e70576-s002.docx]

**Supplementary Figure 1.** Relationship between lactate dehydrogenase–to–albumin ratio (LAR) and Pittsburgh Sleep Quality Index (PSQI) scores

Scatter plot illustrates the association between LAR and PSQI total scores after exclusion of outliers. Linear and logarithmic regression curves are overlaid, demonstrating a strong positive relationship (linear R²=0.72; logarithmic R²=0.73; both p<0.001). This visualization complements the ROC analysis and illustrates the continuous association between LAR and sleep disturbance severity.
